# Dr. Andrew J. Volker

**Published:** 1900-01

**Authors:** 


Dr. Andrew J. Volker, of Buffalo, U. of B., '92, died at his residence in this city, December 11, 1899, aged 28 years. Dr. Volker was a young man of promise and his early death is deeply lamented.

